# Transcriptomic Profile of Human Osteoblast-like Cells Grown on Trabecular Titanium

**DOI:** 10.3390/ijms26083598

**Published:** 2025-04-11

**Authors:** Giovanna Castoldi, Mario Mauri, Deborah D’Aliberti, Silvia Spinelli, Leonardo Testa, Federico Gaverina, Alessandro Rubinacci, Isabella Villa, Giuseppe Bellelli, Gianpaolo Zerbini, Rocco Piazza, Giovanni Zatti

**Affiliations:** 1 Dipartimento di Medicina e Chirurgia, Università degli Studi di Milano-Bicocca, 20900 Monza, Italy; mario.mauri@unimib.it (M.M.); deborah.daliberti@unimib.it (D.D.); s.spinelli22@campus.unimib.it (S.S.); l.testa24@campus.unimib.it (L.T.); f.gaverina@campus.unimib.it (F.G.); giuseppe.bellelli@unimib.it (G.B.); rocco.piazza@unimib.it (R.P.); giovanni.zatti@unimib.it (G.Z.); 2 Clinica Ortopedica, Fondazione IRCCS San Gerardo dei Tintori, 20900 Monza, Italy; 3 Ospedale San Raffaele-Respighi, 20121 Milano, Italy; rubinacci.alessandro@hsr.it; 4 Laboratorio di Endocrinologia e Metabolismo Osseo, Istituto di Scienze Endocrine e Metaboliche, IRCCS Ospedale San Raffaele, 20132 Milano, Italy; villa.isabella@hsr.it; 5 Acute Geriatric Unit, Fondazione IRCCS San Gerardo dei Tintori, 20900 Monza, Italy; 6 Unità Complicanze del Diabete, Diabetes Research Institute, IRCCS Istituto Scientifico San Raffaele, 20132 Milano, Italy; zerbini.gianpaolo@hsr.it; 7 Divisione di Ematologia, Fondazione IRCCS San Gerardo dei Tintori, 20900 Monza, Italy

**Keywords:** human primary osteoblast-like cells, transcriptome, 3D trabecular titanium scaffold

## Abstract

Trabecular titanium implants are widely used in orthopedic surgery and are known to promote osseointegration. In this study, we investigated whether primary human osteoblast-like cells grown inside a 3D trabecular titanium scaffold undergo changes in migration capacity, transcriptomic profile, and cellular phenotype as compared to the same osteoblasts not grown inside the scaffold. Scratch tests have shown that primary human osteoblast-like cells grown inside the 3D trabecular titanium scaffold promote the migration of cells from the external environment into the scaffold. Next generation sequencing analysis demonstrated that primary human osteoblast-like cells grown inside the 3D trabecular titanium scaffold modified the expression of genes involved in cell cycle and extracellular matrix remodeling, while maintaining a normal expression of the specific osteoblast markers, such as osteocalcin and osterix, as well as a comparable mineralization capacity. These data demonstrate that primary human osteoblast-like cells grown inside the titanium scaffold in a 3D environment acquire specific features favoring osseointegration.

## 1. Introduction

The identification of optimal tissue-implant interfaces represents the target of current efforts aimed at improving implant technology for orthopedic applications.

However, the development of a titanium implant that fulfills the wide-ranging requirements of an “ideal” biomaterial is still challenging despite current efforts. The design of an “ideal” biomaterial in bone regeneration is based on the principle of replicating the dynamic architecture of natural bone and providing mechanical support to promote osteogenesis. While titanium shows remarkable mechanical properties as a supportive substrate, the rate of osseointegration of its interface with living bone is highly dependent on its surface properties. Several surface treatment technologies have been developed to attain bioactive surfaces on titanium (Ti) substrates, particularly controlling roughness and oxidation. After the sandblasted and acid-etched process to create titanium surfaces with different roughness, it was indeed evident that the osteoblast proliferation is determined by surface roughness at every oxygen level [1,2], being a surface roughness range between 0.5 and 2 µm at the microscale level, the best interlocking reaction between mineralized bone and implants [3]. It was also shown that a passive oxide layer (TiO2) on the titanium surface contributes to its osteogenic properties, although concerns were raised on possible pathological mechanisms activated by peroxidation products on surface-modified titanium implants [2]. The control on multi-scale surface treatments is therefore critical to enhance osseointegration of the implants and assure their security. As reviewed [3], roughness, chemical composition, and wettability (i.e., the cohesive and adhesive forces that determine the contact of a liquid on a solid surface) constitute the essential cells’ informative parameters, able to differentially activate genome-wide transcriptional responses of osteoblasts [4], thus significantly influencing protein adsorption, and impacting cell attachment. In particular, microrough surfaces modulate numerous cellular functions through molecular mechanisms associated with promoted differentiation, spreading behavior, and intracellular stress response of osteoblasts [4,5,6]. Attempts to improve osseointegration were also devoted to developing interpenetrating phase composites through innovative processing methods aimed at combining the mechanical advantage of titanium with the biological benefit of hydroxyapatite which displays more effective osteo-inductive properties [7]. As discussed [7], this biomimetic process generates a network of concave pores with an optimal size to support human osteoblast proliferation. It has been therefore clear that empowering titanium implants with biological as well as anti-infection properties by multifunction coatings [8] or assembling, layer-by-layer, bone scaffolds to co-deliver bioactive compounds, might enhance osseointegration [9]. Alternative “biomimetics” compounds, as ideal bone grafting material, are still under development [10].

As pioneering experiments have clearly foreseen [2,4], optimal quality and security of the titanium osseointegration process can be predicted and obtained by exploring the transduction pathway of the local bone remodeling signal triggered at the cell/titanium interface by the implanted titanium. The behavior of cells or tissue exposed to different conditions can be indeed evaluated, quantified, and compared by differential expression analysis, an approach that was shown to be effective in different research fields [11,12,13,14].

Since the bone remodeling process, which is the result of a continuous flow of signals and events ensuring functional bone adaptation, is sensitive to both static (3D structural constitution of the bone mineralized matrix) and dynamic (loading of bone mineralized matrix and cells) components of the mechanical environment [15], a better understanding of the bone remodeling signal triggered at the cell/implant interface by the implant itself could be achieved by mimicking more closely the bone structure through an innovative 3D cell culture rather than taking advantage of a classic 2D cell culture, which not fully represents the in vivo mechanical setting. As discussed [16,17], 3D culture models of primary osteoblasts with titanium as well as other scaffold material, might indeed mimic the in vivo conditions more precisely, thus facilitating cell physiological behavior and, consequently, clinical translation.

Along this line of thought, we have therefore designed the following experiment with the direct aim of evaluating whether primary human osteoblasts grown inside a 3D titanium scaffold mimicking trabecular structure modify their transcriptomic profiles, cellular phenotype, and migration capacity when compared to osteoblasts grown outside the scaffold in a 2D cell culture model. By demonstrating that the 3D environment is critical to the modulation of a gene expression profile involved in osteoblast interaction and extracellular matrix remodeling, our results extend the current understanding of bone growth in the scaffold-guided micro-environment and pave the way to targeted modifications of the titanium scaffold architecture to specifically modulate the transduction signal at the implant/tissue interface for optimal support of cell attachment and proliferation.

## 2. Results

### 2.1. Effect on Cell Migration of Osteoblasts-Filled Titanium Scaffold

**Figure 1:**
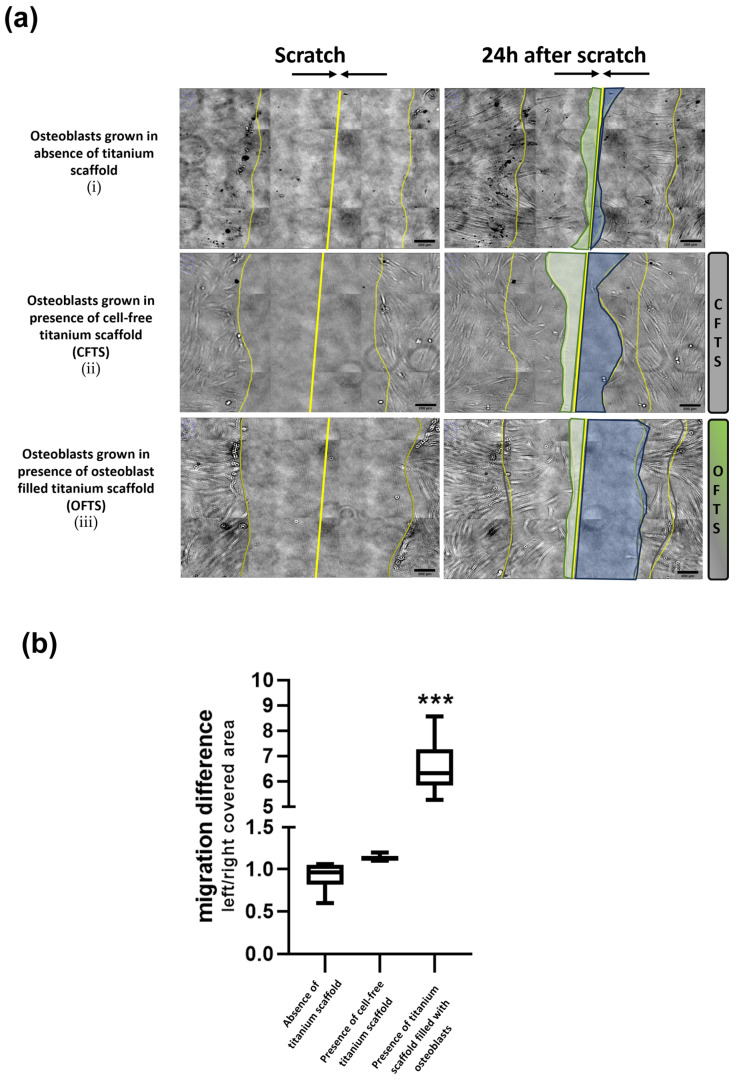
Migration experiments. (**a**) Representative image of wound healing after scratch test in osteoblasts grown in the absence of titanium scaffold (**i**), in the presence of cell-free titanium scaffold (**ii**), and in the presence of titanium scaffold filled with osteoblasts (**iii**). The edges of the scratch and the relative center are highlighted in yellow. In the right images, the light green and light blue regions represent the uncovered area after 24 h of recorded migration. Scale bar: 200 µm (**b**) Quantification of the migration index calculated with the reported formula: Δmigration = covered left area/covered right area. Each box plot shows the median and extends from the lowest to the highest value (*n* = 7 independent experiments). *** *p* < 0.001 vs. other conditions, two-way ANOVA with Tukey’s correction for multiple comparisons.

Results obtained from the migration experiments are shown in Figure 1a,b. Scratch tests performed in each donor considered for the study demonstrated that after 24 h, osteoblasts seeded directly into the well containing cell-free titanium scaffold (Figure 1a(ii)) migrated from left to right (toward titanium scaffold) and from right to left (trying to close the empty space) in a way that was substantially similar to the one implemented by osteoblasts seeded in the absence of titanium scaffold (Figure 1a(i)). These pieces of evidence indicate that the presence of a titanium scaffold does not influence the migration characteristics of osteoblasts.

Conversely, when osteoblasts are seeded in the presence of the titanium scaffold and actually grow inside it, the left-to-right migration toward the titanium scaffold in response to scratch (Figure 1a(iii)) is greater than the one from right to left (Figure 1a(iii)), possibly suggesting that, when grown within the scaffold, osteoblasts might become able to modify their transcriptome profile and cellular phenotype.

### 2.2. Primary Osteoblasts Grown Inside Titanium Scaffold Maintain Osteoblast Phenotype, Mineralization Capacity, and Increase Collagen-1A1 Expression

**Figure 2:**
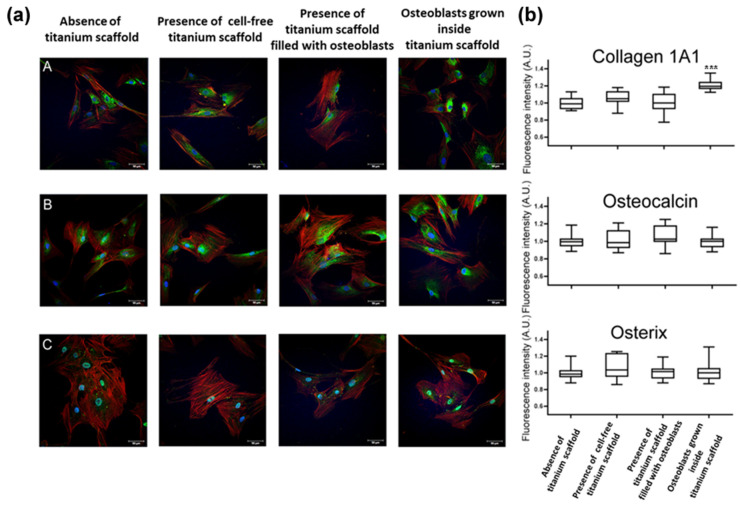
Osteoblast marker expression. (**a**) Representative images of osteoblasts labeled with different antibodies (Green signal) against Collagen 1A1 (**A**), Osteocalcin (**B**), and Osterix (**C**), counterstained with phalloidin for actin fibers (Red signal) and DAPI for nuclear staining (Blue signal) in osteoblasts grown in the absence of titanium scaffold, in osteoblasts grown in the presence of cell-free titanium scaffold, in osteoblasts grown in the well in the presence of a titanium scaffold filled with osteoblasts; in osteoblasts grown inside and extracted from the titanium scaffold. Scale bars: 50 μm. (**b**) Confocal microscopy quantification of Green Integrated Fluorescence Intensity. Each box plot shows the median and extends from the lowest to the highest value (*n* = 3 independent experiments). *** *p* < 0.001 vs. other conditions, two-way ANOVA with Tukey’s correction for multiple comparisons.

Figure 2a,b shows representative immunofluorescence images and the quantification of immunofluorescence staining for Collagen-1A1, Osteocalcin, and Osterix expression in osteoblasts in the different experimental conditions. For each donor, osteoblasts grown inside a titanium scaffold expressed osteoblast marker osteocalcin and the transcription factor osterix equally as osteoblasts grown in the absence of a titanium scaffold, the presence of cell-free titanium scaffold, or the presence of a titanium scaffold filled with osteoblasts. On the contrary, the osteoblasts seeded and grown inside the titanium scaffold, when extracted and re-seeded in a 2D domain, showed an increase in the cytoplasmatic expression of collagen-1A1 compared to the other conditions, suggesting that, while maintaining the osteoblastic phenotype, cells grown inside titanium scaffold promote matrix synthesis.

**Figure 3:**
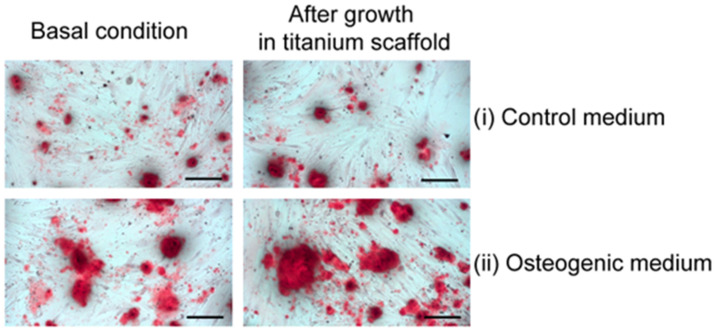
Mineralization capacity. Representative images of osteoblasts before and after growth in titanium scaffold maintained in control medium (Iscove) or stimulated with osteogenic medium (Omem) and finally stained with alizarin red. Increased alizarin red staining was detectable in osteoblast culture grown in osteogenic medium (**ii**) compared to those grown in standard Iscove culture medium (**i**). Osteoblasts grown into the titanium scaffold maintained mineralization capacity as those that have never encountered titanium scaffold. Scale bar: 50 μm.

To evaluate whether the osteoblasts grown inside the 3D trabecular titanium scaffold maintained the capacity to mineralize the matrix produced by themselves, alizarin red staining, which detects the calcium deposits, was performed. Figure 3 shows for each patient studied that osteoblasts collected from a titanium scaffold stimulated with osteogenic medium (Omem) for 28 days retained the ability to mineralize as observed in the osteoblasts grown outside the titanium scaffold (Figure 3).

### 2.3. Primary Osteoblasts Grown Inside Titanium Scaffold Modify Their Transcriptomic Profile

**Figure 4:**
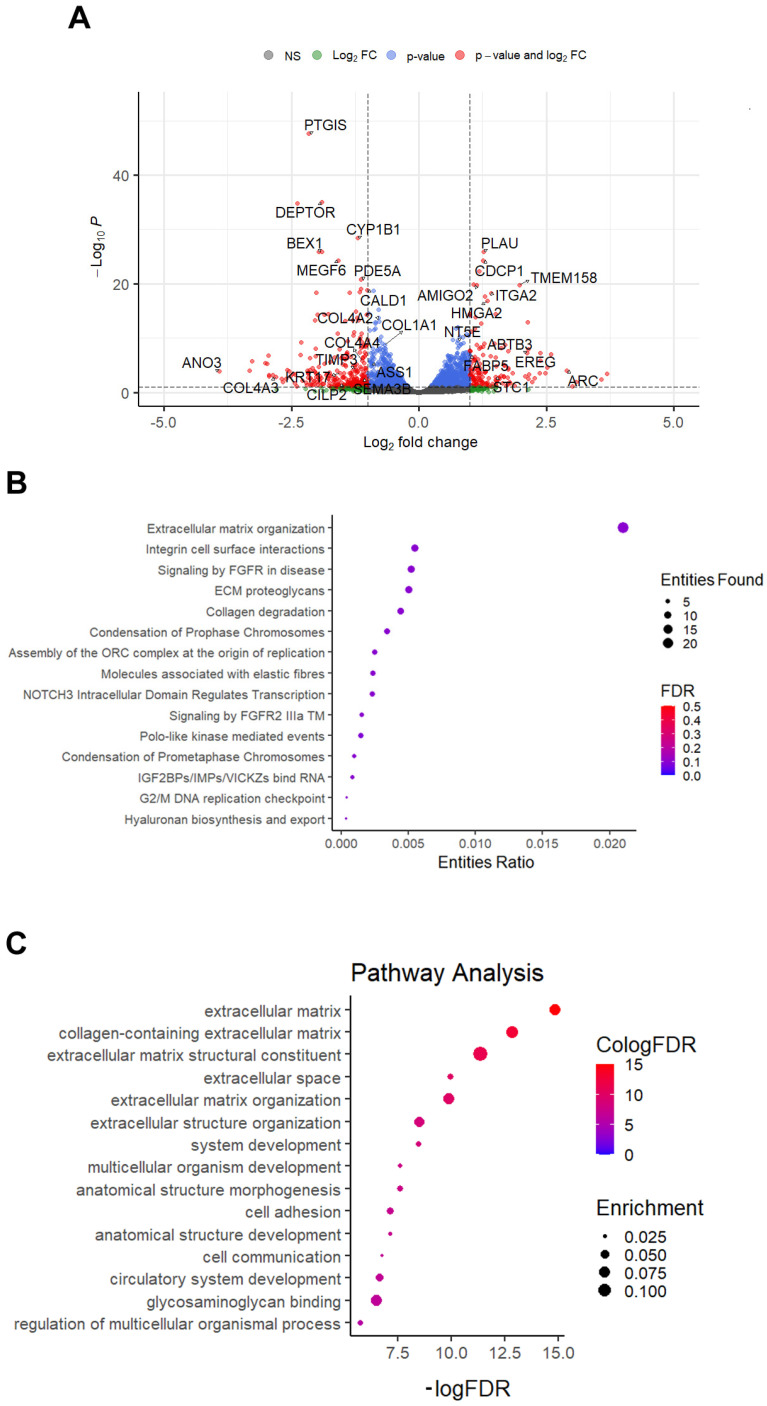
Differential expression analysis of osteoblasts extracted from the 3D trabecular titanium scaffold vs. osteoblasts grown in 2D in the presence of a filled 3D trabecular titanium scaffold. (**A**) Volcano Plot reporting the results of the differential analysis; red dots represent statistically differential genes characterized by an absolute Log_2_ Fold-Change > 1; blue dots represent statistically differential genes characterized by an absolute Log_2_ Fold-Change < 1. (**B**,**C**) CPDB (**B**) and Reactome (**C**) pathway over-representation analysis of osteoblasts extracted from 3D trabecular titanium scaffold vs. osteoblasts grown in 2D in the presence of filled 3D trabecular titanium scaffold. Dots represent individual pathways. Dot size is proportional to the number of differential genes present in each pathway.

Taken globally, these data suggest a specific role for the 3D trabecular titanium scaffold in modulating osteoblast motility and extracellular matrix remodeling. To further characterize these data, we performed whole-transcriptome sequencing on osteoblasts grown in the four conditions: (1) absence of scaffold (Control), (2) presence of a cell-free 3D trabecular scaffold (Empty), (3) presence of an osteoblast-filled 3D trabecular scaffold (Filled), (4) osteoblasts extracted from the 3D trabecular titanium scaffold (Titanium). No evidence of differentially expressed transcriptional programs could be found in the Empty vs. Control, Filled vs. Control, and Filled vs. Empty contrasts (see Tables Supplemental Materials: Comparison between osteoblast differential gene expression analysis in the different conditions: Empty vs. Control; Filled vs. Control; Filled vs. Empty). On the opposite, the Titanium vs. Filled (differential expression analysis revealed the presence of 424 differentially expressed genes (DEGs) (Figure 4A) (see Tables Supplemental Materials: Comparison between osteoblast differential gene expression analysis in the different conditions: Titanium vs. Filled). Over-representation analysis carried out on the 424 DEGs using CPDB (ConsensusPathDB-human) (Figure 4B) and Reactome (Figure 4C) revealed enrichment for pathways associated with extracellular matrix (ECM) organization, integrin cell surface interaction, ECM proteoglycans, collagen turnover, and cell proliferation.

**Figure 5:**
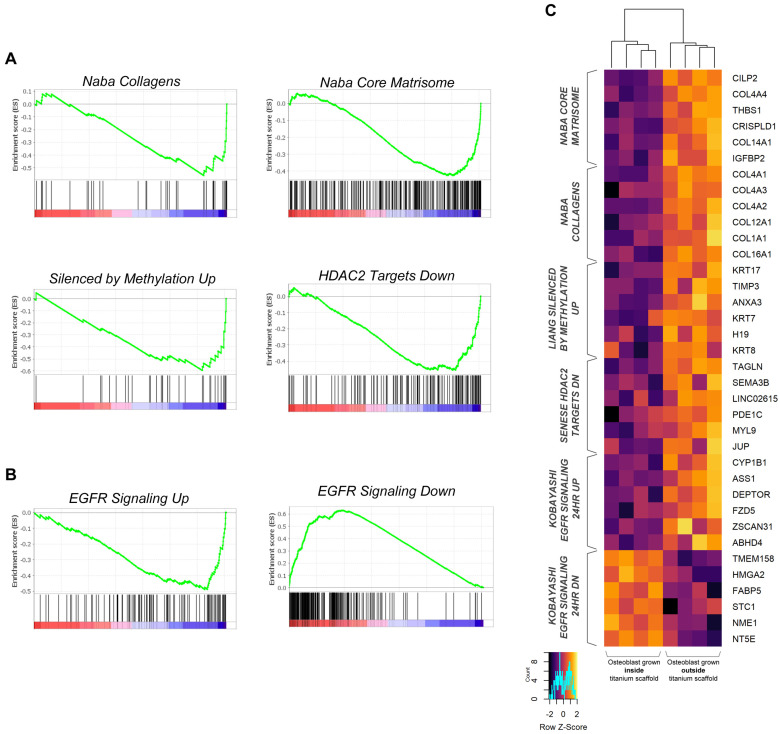
Gene Set Enrichment Analysis (GSEA) of osteoblasts extracted from the 3D trabecular titanium scaffold vs. osteoblasts grown in 2D in the presence of a filled 3D trabecular titanium scaffold. (**A**) Gene Sets associated with extracellular matrix remodeling and epigenetic regulation. (**B**) Gene Sets associated with EGFR activity. (**C**) Heatmap reporting the top enriched genes identified in the GSEA analyses shown in (**A**,**B**).

Gene Set Enrichment Analysis (GSEA) carried out on osteoblasts extracted from the 3D trabecular titanium scaffold genes vs. osteoblasts grown in 2D in the presence of a filled 3D trabecular titanium scaffold, showed a negative enrichment for the NABA_CORE_MATRISOME (Figure 5A,C, Supplemental Figure S1), a reference group of genes encoding core extracellular matrix including ECM glycoproteins, collagens and proteoglycans, and the NABA_COLLAGENS (Figure 5A,C, Supplemental Figure S2) gene sets. These findings support the notion that osteoblasts grown inside the titanium scaffold modulate key pathways responsible for extracellular matrix remodeling. In the effort to investigate the specific mechanisms by which the presence of the scaffold filled with osteoblasts could modify the expression of genes of extracellular matrix pathways, as well as the increase in the migratory potential of nearby osteoblasts, we also noticed a significant involvement of several pathways associated with epigenetic modulation. In particular, the *LIANG_SILENCED_BY_METHYLATION_UP* gene set (Figure 5A,C, Supplemental Figure S3), comprising genes up-regulated in fibroblasts after treatment with the DNA hypomethylating agent decitabine, as well as the SENESE_HDAC2_TARGETS_DN (Figure 5A,C, Supplemental Figure S4) comprising genes down-regulated upon knockdown of HDAC2, were both negatively enriched in osteoblasts grown in the scaffold, suggesting that the modulation of the cell phenotype could be at least in part due to the activation of epigenetic remodulation pathways skewed towards methylation/deacetylation and gene silencing.

Finally, GSEA carried out on osteoblasts extracted from the 3D trabecular titanium scaffold genes vs. osteoblasts grown in 2D in the presence of a filled 3D trabecular titanium scaffold highlighted a very strong positive enrichment of the EGFR signaling pathway (Figure 5B,C, Supplemental Figure S5), suggesting a pivotal role of EGFR in driving osteoblast migration. In line with these findings, epiregulin (EREG), a ligand of the epidermal growth factor receptor involved in a wide range of biological processes such as wound healing and cell proliferation, was found to be upregulated in osteoblasts extracted from the 3D trabecular titanium scaffold (Figure 4A).

## 3. Discussion

The results of this study demonstrate that human primary osteoblasts, obtained from matched donors of both genders, when grown inside a 3D trabecular titanium scaffold modify their transcriptomic profiles and migration capacity as compared to the same cells, derived from the same donors, but grown in 2D culture, both in the presence or absence of the 3D trabecular titanium scaffold.

The analysis of the whole-transcriptome data, done to explore the osteoblasts transduction pathway triggered by the titanium scaffold, revealed that the osteoblasts grown inside the 3D micro-environment, constituted by the scaffold itself, showed a selective enrichment of the cell pathways associated with the cell cycle, EGFR signaling, extracellular matrix organization, and turnover.

Despite these modifications, the primary characteristic of the osteoblasts, as their mineralization capacity, is preserved in the cells grown inside the scaffold. Also, the osteoblastic markers (osteocalcin and osterix) are similarly expressed in the osteoblasts grown inside the 3D trabecular titanium scaffold and in those grown outside the scaffold in a 2D domain.

We used a 3D scaffold made in biomedical Ti6AI4V powder, in accordance with ASTM F3001 standard, since this biomaterial is usually applied in orthopedic surgery for its high biocompatibility, chemical stability, and tensile strength matching the mechanical properties of bone. Its 3D structure was produced by Additive Manufacturing (AM), using Electron Beam Melting Technology (EBM). The Computer-Aided Design (CAD) of the 3D printed model mimicked the trabecular distribution of the human bone with accurately designed pore parameters and with a porosity of 64 percent. The precise control of the 3D environment, obtained by CAD, favors physiological cell adaptation [18] by providing an adequate mechanical environment for osteoblast growth [19], viability [20], and activity [21].

The 3D domain is indeed a critical aspect of osseointegration since it mimics the complex multiscale architecture of bone [22,23] and better recapitulates its spatial organization. By allowing cells to interact along different planes [24], the 3D domain establishes a privileged environment vs. 2D cultures for osteoblasts differentiation, mineral deposition [25], communication [26], maturation toward osteocytes [27], and finally osteogenesis [28].

Our study strongly supports this notion by showing that the 3D domain of the scaffold is the pivotal environmental signal involved in shaping osteoblast gene transduction signature at the cell/titanium interface and inducing osteoblast functional changes. In fact, human primary osteoblasts experiencing a 3D domain during growth, i.e., osteoblasts extracted from the scaffold, retained the ability to mineralize but were the only ones showing modified migration capacity, collagen production, and the differential expression of 424 genes. On the contrary, osteoblasts grown in the presence of the titanium scaffold, filled or not with osteoblasts, in a 2D domain did not show any difference in the cell phenotype from the osteoblast grown in the absence of the titanium scaffold, and did not activate a specific transcriptomic profile.

The analysis of their transcriptional profile has shown the concomitant modulation of the genes involved in the pathway of cell communication and integrin cell surface interactions that regulate cell adhesion events (Figure 4, panel B). Interestingly, many genes involved in the EGFR signaling pathway, a key pathway involved in cell proliferation, known to be positive regulators of cell migration via the modulation of actin fibers dynamics [29], were positively modulated in the osteoblasts grown inside the 3D trabecular titanium scaffold (Figure 5, panel B). Since the EGF/EGFR signaling pathway also plays an important role in bone development and metabolism [30,31,32], it is reasonable to speculate that the increased migratory potential of osteoblasts towards the 3D trabecular titanium scaffold filled with osteoblasts (Figure 1a(iii),b) might be linked to the modulation of this pathway. It is also reasonable to hypothesize that the presence of osteoblasts in the scaffold can act as a decoy for external cells, perhaps through the secretion of chemoattractant factors. It is also interesting to note that epiregulin (EREG), a member of the epidermal growth factor family, encoding a protein that is a ligand of epidermal growth factor receptor, is upregulated in osteoblasts extracted from the 3D trabecular titanium scaffold.

Although further studies will be required to thoroughly dissect the molecular bases of this phenomenon and to identify the specific link between the presence of the scaffold filled with osteoblasts and the activation of the EGFR signaling pathway, our results are in line with the view that 3D topography of surrounding environment might activate cytoskeleton crosstalk [33].

Topography has emerged as a discriminating stimulus regulating the biological phenomena engaging morphogenesis, cell migration, and differentiation, and its mediated effects convey the great advantage of being more stable than a chemical stimulus carried by the biomaterial coating that can be biodegradable [34]. How osteoblasts sense “*non ordered topography*” under nano-micro-scales (i.e., roughness), as well as “*ordered topography*” under macro-scale (i.e., shape, dimension, orientation, periodicity of interconnected pores), as recently defined [34], is a matter of current interest, based on the observation that osteoblast precursors shape, as modulated by targeted modification of the micro- and macro-structures of the scaffold to which they adhere, may control osteoblast fate [35]. As reviewed by Rougerie et al. [34], the topographical information might be subsequently transduced by several molecular mechanisms involving BMP receptors [36], activating Smad Family transcription factors also inducing osteoblastogenesis and bone formation-related genes, such as RUNX2 and OSX, as well as WNT pathway regulating osteoblasts’ maturation [37]. However, the modulation of the integrin-mediated adhesion processes in response to a specific topography, such as the 3D domain in our experiment, appears to be a pivotal pathway in the transduction of the topographic signal. The osteoblast transcriptomic profile outlined in our experiment (Figure 4 panels B and C) fits this view but also suggests that the EGFR pathway, as discussed above, might be an additional transcription mechanism of the topographic signal.

An intriguing result of our study is also related to the modulation of genes involved in bone development and homeostasis, such as genes belonging to fibroblast growth factor (FGF)/fibroblast growth factor receptor (FGFR) signaling [38,39,40]. In particular, the transcriptomic profile reported in Figure 4, panel B, revealed the specific activation of the FGFR2 signaling. As discussed [41], FGFR2 interacts with FGF2, the most common ligand of the FGF family that gained attention in regenerative medicine. FGF2 indeed is an endogenous, positive regulator of bone mass that enhances osteogenesis [42,43].

Interestingly, genes involved in cell growth, apoptosis, and differentiation, such as high mobility group AT-hook 2 (Hmga2), which is differentially involved in osteoblast differentiation [44,45] and bone growth [44], or apoptosis repressor with caspase recruitment domain (ARC), which play a role in the regulation of apoptosis and osteogenic differentiation [46,47], or DEP domain containing mTOR interacting protein (DEPTOR), a natural mTOR inhibitor with a role in transcriptional regulation [48] and in bone disease [49,50], are differentially expressed in osteoblasts growth inside the 3D trabecular scaffold as compared with osteoblasts grown in 2D domain, showing a complex modulation of gene transcription.

In conclusion, the human primary osteoblasts, extracted from the scaffold and experiencing a 3D domain during growth, retained the ability to mineralize but showed different migration capacities, collagen production, and 424 differential expressed genes vs. osteoblasts grown in a 2D domain. The analysis of the transcriptomic profile of human primary osteoblasts outlined in our study has enhanced the current understanding of how the 3D topographic information carried by the specific architectures of the selected scaffold is transduced at the cell level. By demonstrating the modulation of genes encoding cell adhesion, integrin interaction, cell communication, and extracellular matrix organization, we outlined potential pathways to empower osteoblast interactions and the subsequent colonization of the scaffold itself. Although our study is at an early stage, a first biologically relevant outcome might be envisaged and related to the possibility of drawing the molecular background of osseointegration, once the essential cells’ informative parameters have been defined. They not only depend on the surface characteristics of the titanium substrate, as generally acknowledged, but on their interacting role with the 3D printing of the scaffolds with favorable pore architectures of the trabecular network. We think that a deeper analysis of how the identified differentially expressed genes could be targeted or manipulated in future therapies to accelerate the osseointegration process is premature since the analysis of the cells’ responses to specific and interacting macro- and micro-topographic characteristics of the scaffold is still underway. Nevertheless, the approach applied in our study could allow the recognition of the main signals, intrinsic to the scaffold itself, i.e., the so-called “*topographical code*” [34], that can specifically target the genes favorable to the osteoblast adaptation at the scaffold/cell interface with potential clinical application.

## 4. Materials and Methods

### 4.1. Titanium Scaffolds

Three-dimensional trabecular titanium scaffolds (REF DCI-B000) made in biomedical Ti6Al4V ELI (Extra-Low Interstitial) in accordance with ASTM F3001 were a generous gift of the manufacturer Gruppo Bioimpianti Srl (Peschiera Borromeo, MI, Italy). The 3D structure was produced by Additive Manufacturing (AM) using Electron Beam Melting technology (EBM) and starting from Ti6Al4V ELI powder. The Computer-Aided Design, CAD, on a 3D printed model, mimicked the trabecular distribution of the human bone with accurately designed pore parameters. The 3D trabecular titanium scaffolds were characterized by a selected porosity of 64% and by a Diamond basic trabecular cell unit randomly distributed. The estimated pore size was 300–800 µm taking into account the random distribution of the basic trabecular cell units.

### 4.2. Primary Human Osteoblast Cultures

Primary human osteoblast cultures were obtained from the waste material of female and male donors during orthopedic surgery for degenerative diseases or traumatic fractures of the femoral neck. The donors (aged 68–89 yrs, nine females, five males) signed the informed consent for the use of the waste material, and the protocol (Reference number 3505, 6.1 EME1-25.5.23) was approved by the Ethical Committee of IRCCS San Gerardo dei Tintori di Monza (Italy). None of the donors was affected by infective disease and malignancies. Briefly, the trabecular bone was cut into small pieces, rinsed, and incubated with rotation at 37 °C for 30 min with 0.5 mg/mL type IV collagenase. The bone pieces were then placed in 25 cm^2^ flasks and cultured in Iscove’s modified medium (IMDM) containing 10% FBS, 100 U/mL penicillin, 100 μg/mL streptomycin, and 0.25 μg/mL amphotericin B until confluence. The medium was changed twice a week. Cells were used at the first passage to reduce the possibility of phenotype changes [51].

### 4.3. Experimental Design

**Figure 6:**
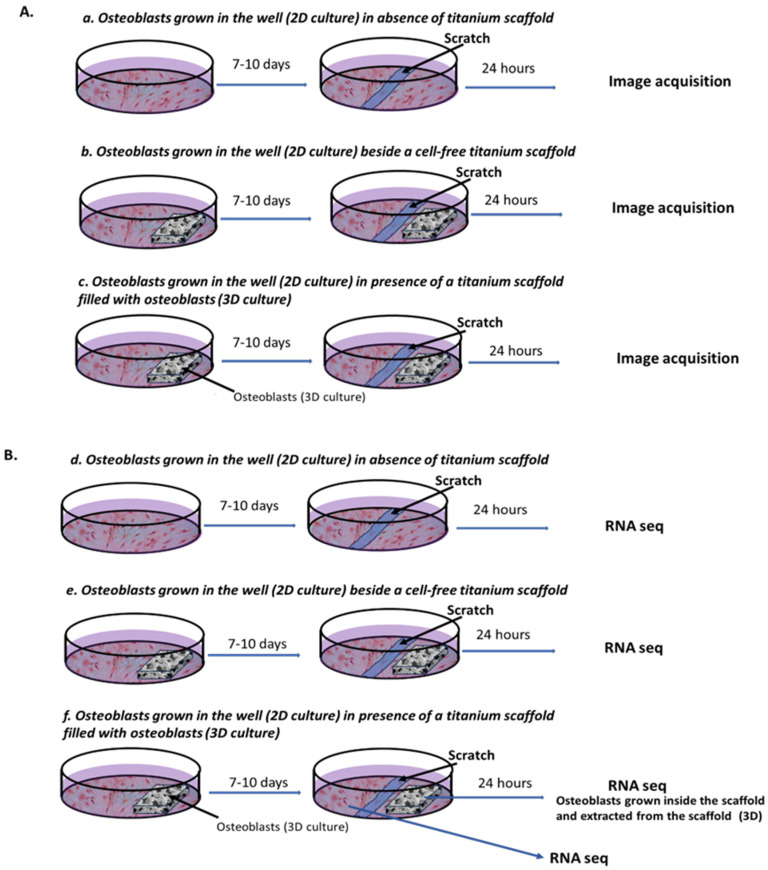
Schematic representation of experimental design for scratch test experiments and RNAseq analysis. Osteoblasts were seeded in the absence of a 3D trabecular titanium scaffold (**a**,**d**), with a cell-free 3D trabecular titanium scaffold (**b**,**e**) or with a 3D trabecular titanium scaffold with osteoblasts seeded inside it (**c**,**f**). At confluence, after 7–10 days, a scratch test was performed to evaluate 24 h osteoblast migration (**A**) and to collect samples for RNAseq (**B**).

Figure 6A,B schematize the main steps and the timeline of the experimental protocol. For each sample, human osteoblasts were cultured in the first lane of a six-well culture plate in order to have a well without a 3D trabecular titanium scaffold (a.,d.), and two wells with 3D trabecular titanium scaffold fixed on the base (b.,c.,e.,f.).

The cells were seeded in the well without a 3D trabecular titanium scaffold (a.,d.) and in a well with a 3D trabecular titanium scaffold directly at the layer of the well (b.,e.). In the other well with a 3D trabecular titanium scaffold, the cells were placed directly on the titanium scaffold (c.,f.), and after initial attachment (30 min at 37 °C, CO_2_ 5%), the culture medium was added.

The cells were cultured in a humidified atmosphere at 37 °C, CO_2_ 5% until confluence (7–10 days). Once the osteoblasts reached confluence, a tip was used to trace a groove (scratched area) in the middle of the well without a titanium scaffold (a.,d.) and immediately next to the 3D titanium scaffold (b.,c.,e.,f.). After washing with PBS (Sigma, Darmstad, Germany), the osteoblasts were cultured for 24 h under an inverted widefield microscope (CellObserver Zeiss, Jena, Germany) using a 20× objective (0.5AN) with a phase contrast ring (Figure 6A). Acquisition of different conditions was performed using a motorized stage ensuring a parallel recording of different wells.

Cells were imaged every 10 min for 24 h and results were analyzed with a home-designed macro in ImageJ (version 1.54f). Briefly, in the first acquired image we measured the scratched area, and in the last one we measured the uncovered area making a ratio between the scratch edges and the center of the wound (Figure 1).

For RNA-seq transcriptional analysis, the osteoblasts of four donors (two females and two males) were seeded at the same time in two six-well plates in the first lanes and underwent the same scratch test procedure as described alone. The cells of one plate were used for the registration of 24-hr migration (Figure 6A). After 24 h, the cells from the other plate were washed with PBS and the cells were collected in Trizol reagent (Invitrogen, Carlsbad, CA, USA) from osteoblasts grown in the absence of titanium scaffold (d.), and from osteoblasts grown beside cell-free titanium scaffold (e.), after removing the 3D trabecular titanium scaffold. From the 3D trabecular titanium scaffold on which the cells were seeded (f.), the cells were harvested separately from the titanium scaffold (osteoblast grown into 3D trabecular titanium scaffold) and from the osteoblasts grown in the well (f.). Furthermore, for each patient studied, four samples representing the four different experimental conditions—osteoblasts grown in 2D culture in the absence of titanium scaffold (Control); osteoblasts grown in 2D culture in the presence of cell-free 3D trabecular titanium scaffold (Empty); osteoblasts grown in 2D culture in the well in the presence of a 3D titanium scaffold filled with osteoblasts (Filled); osteoblasts extracted from the 3D trabecular titanium scaffold (Titanium)—were collected and stored at −80 °C until RNA extraction.

### 4.4. Immunofluorescence Analysis and Alizarin Stain

To evaluate whether osteoblasts grown in a titanium scaffold maintain osteoblast phenotype, primary osteoblasts were seeded and grown inside a titanium scaffold in six-well culture plates. The culture medium was changed twice a week until osteoblasts in the well containing the scaffold reached the confluence (10–15 days). Then the scaffold was removed and placed in trypsin (Sigma) in a 50 mL Falcon tube and centrifuged at 1700× *g* for 10 min. The pellet was then washed and resuspended in a medium and osteoblasts were seeded for 5–7 days for immunofluorescence analysis to evaluate osteocalcin, osterix, and collagen 1A1.

For the four different experimental conditions (osteoblasts grown in the absence of titanium scaffold; osteoblasts grown in the presence of cell-free titanium scaffold; osteoblasts grown in the presence of 3D trabecular titanium scaffold filled with osteoblasts; osteoblasts grown inside titanium scaffold), osteoblasts were fixed with paraformaldehyde 4% and stored at 4 °C until immunofluorescence analysis. A primary polyclonal antibody against osteocalcin (PA5-96529, Thermo Fisher Scientific, Waltham, MA, USA), osterix (PA5-115697, Thermo Fisher Scientific, Waltham, MA, USA), and collagen 1A1 (PA5-29569, Thermo Fisher Scientific, Waltham, MA, USA ) was added and the samples were incubated for 2 h at room temperature. After three washes with PBS, the samples were incubated with Alexa 488 conjugated antibody for 90 min.

Images were acquired using a Zeiss LSM 710 confocal laser-scanning microscope (Zeiss, Jena, Germany) using a 63×, 1.4 N/A oil-immersion objective. Laser intensities and acquisition parameters were held constant throughout each experiment.

Confocal microscopy fields were analyzed using a specific homemade-designed macro with ImageJ software. In detail, COL1A1, osterix, and osteocalcin signal intensity were analyzed by measuring the integrated density (ID) and normalized over the signals’ recorder on cells cultured in the control condition. All the data obtained was derived from at least 10 fields for experimental conditions (at least 80 cells each).

Alizarin red (A5533. Sigma-Aldrich, St. Louis, MO, USA) was used to evaluate the presence of calcium in the extracellular matrix. To evaluate mineralization, osteoblasts from the same patient (*n* = 3) were stimulated in a differentiation medium (OMEM, for 28 days) before (basal conditions) and after being grown in titanium. Osteoblasts harvested from the titanium scaffold were seeded into two well plates. After reaching confluence, the medium was removed, cells were washed with PBS and a new Iscove medium was added to one well, while a differentiation medium (OMEM) was added to the other well for 28 days. The cells were then fixed in 4% paraformaldehyde for 10 min and then washed twice with distilled water before staining with 1% alizarin red solution for 30 min in oscillatory sketching [52]. Samples were washed five times with distilled water and analyzed with an inverted widefield microscope equipped with a CCD color camera.

### 4.5. RNA Extraction, Sequencing, and Data Analysis

RNA from osteoblasts was extracted using the Trizol Reagent (Invitrogen) according to the manufacturer’s instructions. RNA libraries were prepared using SMARTer Stranded Total RNA-Seq Kit v2—Pico Input Mammalian Kit (Takara Bio USA). The integrity of the RNA samples was assessed using a TapeStation (Agilent Technologies, Santa Clara, CA, USA). A total of 10 ng of total RNA was fragmented and converted to cDNA through a reverse transcription reaction. Barcoded adapters for Illumina sequencing were added through polymerase chain reaction (PCR) and the PCR products were purified using AMPure XP beads (Beckman Coulter). Library fragments originating from ribosomal RNA (rRNA) and mitochondrial RNA (mtRNA) were depleted using probes specific to mammalian rRNA and human mtRNA. The cDNA fragments were further enriched in a second round of PCR using universal primers and the amplification products were purified once more to yield the final cDNA library. Libraries profile and concentration were assessed by running samples on a TapeStation (Agilent Technologies). Final libraries were quantified using a Qubit fluorometer (Thermo Fisher Scientific).

RNA-Seq libraries were sequenced using an Illumina NovaSeq 6000, using a 150x2 paired-end design, generating an average of 40 million uniquely mapped reads per sample. Following an initial quality check using FastQC (https://www.bioinformatics.babraham.ac.uk/projects/fastqc/ (accessed on 6 April 2025 )), raw reads were aligned using the human GRCh38/hg38 genome as a reference. The alignment was carried out with the splice-aware aligner Star [53] by applying the quantMode GeneCounts parameter, in order to generate per-gene read counts. Indexing of Bam files was performed using Samtools [54]. The sorted, indexed bam alignment files, were manually inspected using the Integrative Genomics Viewer [55].

Differential expression analysis was carried out with DESeq2 v. 1.30 [56] using a dedicated design model focused on the specific contrast while controlling for the individual genotypes and raw counts as input. Pre-filtering was applied to keep only rows with ≥1 count in all samples. Differential genes were identified by applying a Benjamini-Hochberg corrected *p*-value threshold of 0.1 to control for false positives. Independent filtering was performed by setting the alpha threshold to 0.1. GSEA analyses were carried out with the GSEA software v. 4.2.1 using 1000 rounds of permutation, a Signal2Noise metric, and a weighted scoring scheme. Gene sets were considered significantly enriched in the presence of a Benjamini-Hochberg corrected *p*-value < 0.25. Priority was given to gene sets characterized by a higher absolute Normalized Enrichment Score (NES). Gene set-associated pathways were generated using the ClueGO (v.2.5.10) Cytoscape (v.3.10.3) application, by selecting the Reactome Pathway dataset, medium network specificity, and filtering for pathways with associated *p*-value < 0.05. Over-representation analysis was carried out using CPDB (cpdb.molgen.mpg.de) and Reactome (https://reactome.org/). In both cases pathways were considered enriched in the presence of a Benjamini-Hochberg corrected *p*-value < 0.1.

## Data Availability

The gene expression data presented in this study are available at the end of the embargo at the following link: https://www.ncbi.nlm.nih.gov/sra/PRJNA1229826. The other data presented in this study are available on request from the corresponding author.

## Supplementary Materials

The supporting information can be downloaded at: https://www.mdpi.com/article/10.3390/ijms26083598/s1.

## Abbreviations

The following abbreviations are used in this manuscript:

Ti	titanium
ECM	extracellular matrix
DEG	differentially expressed genes
GSEA	gene set enrichment analysis
CPDB	ConsensusPathDB-human
HDAC2	histone deacetylase 2
EGFR	epidermal growth factor receptor
EGF	epidermal growth factor
EREG	epiregulin
BMP	bone morphogenetic protein
RUNX2	runt-related transcription factor 2
OSX	osterix
WNT	wingless-related integration site family
FGF	fibroblast growth factor
FGFR	fibroblast growth factor receptor
